# Safety and immunogenicity of Rift Valley fever MP-12 and arMP-12ΔNSm21/384 vaccine candidates in goats (*Capra aegagrus hircus*) from Tanzania

**DOI:** 10.4102/ojvr.v86i1.1683

**Published:** 2019-01-31

**Authors:** Salama Nyundo, Ester Adamson, Jessica Rowland, Pedro M. Palermo, Mirende Matiko, George E. Bettinger, Philemon Wambura, John C. Morrill, Douglas Watts

**Affiliations:** 1 Department of Microbiology, Parasitology and Biotechnology, Sokoine University of Agriculture, Tanzania; 2 Department of Biological Sciences, University of Texas at El Paso, United States; 3 Orion Research and Management Services, Texas, United States

## Abstract

Vaccination of domestic ruminants is considered to be an effective strategy for protecting these animals against Rift Valley fever (RVF), but available vaccines have limitations. Therefore, the aim of this study was to determine the safety and immunogenicity of RVF virus (RVFV) mutagenesis passage 12 (MP-12) and arMP-12ΔNSm21/384 vaccine candidates in goats (*Capra aegagrus hircus*) in Tanzania. Goats were vaccinated intramuscularly with RVFV MP-12 or arMP-12ΔNSm21/384, and then on Day 87 post-vaccination (PV) all animals were revaccinated using the RVFV MP-12 vaccine candidate. Serum samples were collected from the animals before and after vaccination at various intervals to test for RVFV using a Vero cell culture assay and reverse transcription polymerase chain reaction and for RVFV-neutralising antibody using a plaque reduction neutralisation assay. Serum samples collected before vaccination on Days -14 and 0, and on Days 3, 4 and 5 PV were negative for RVFV and neutralising antibody. All animals remained healthy, and viremia was not detected in any of the animals. Rift Valley fever virus antibody was first detected on Day 5 PV at a 1:10 dilution in five of five animals vaccinated with the MP-12 vaccine and in five of eight animals vaccinated with arMP-12ΔNSm21/384. Titres then increased and were sustained at 1:40 to 1:640 through to Day 87 PV. All animals that were revaccinated on Day 87 PV with MP-12 developed antibody titres ranging from 1:160 to as high as 1:10 240 on Days 14 and 21 PV. Although the antibody titres for goats vaccinated with RVF MP-12 were slightly higher than titres elicited by the arMP-12ΔNSm21/384 vaccine, these findings demonstrated that both vaccines are promising candidates for the prevention of RVF among Tansanian goats.

## Introduction

Rift Valley fever (RVF) is an important zoonotic disease in Africa and the Arabian Peninsula affecting both humans and animals, especially domestic ruminants (Balkhy & Memish 2003; Pepin et al. 2010). The disease is caused by the RVF virus (RVFV), a negative single-stranded RNA virus that belongs to the order Bunyavirales, family Phenuiviridae, genus *Phlebovirus* (Rima et al. 2017). The disease in animals is characterised by fever, ocular and nasal discharge, bloody diarrhoea, abortion storms in gestating ewes and 90% – 100% mortality in newborn lambs. In humans, the disease causes self-limiting febrile illness, but in about 1% – 2% of cases clinical symptoms progress to neurological disorder, vision loss, haemorrhagic fever and even death (Madani et al. 2003). The disease was first identified during an epizootic and epidemic among sheep and humans on a farm in 1931 in the Rift Valley of Kenya (Munyua et al. 2010). Subsequent outbreaks have been reported from numerous countries throughout Africa and the Arabian Peninsula. Outbreaks in East African countries usually occur following heavy rainfall that results in an increase in the abundance of mosquito vectors. In Tanzania, outbreaks occur every 5–15 years with low-level transmission of RVFV between outbreaks (Sumaye et al. 2013; Woods et al. 2002). The first outbreak in Tanzania was documented in 1977 and the most recent one occurred during 2006–2007 (Anyamba et al. 2010; Jost et al. 2010). In contrast to the most recent outbreak that affected humans and livestock in 52.4% of the regions in Tanzania, previous outbreaks only affected livestock primarily in the northern parts of the country (Faburay et al. 2017).

As a result of the devastating impact of RVF on human and animal health in Tanzania and other RVFV-enzootic countries, several vaccines have been developed, and some are currently being used in an attempt to prevent this disease among livestock in Africa (Faburay et al. 2017). Vaccines offer the most promising control and prevention strategy for RVF because they can afford protection by inducing humoral and cell-mediated immune responses, as well as by enabling vaccinated animals to transfer colostrum that contains maternally acquired antibody to their offspring. (Dar et al. 2013; Labeaud, Kazura & King 2010; Morrill et al. 1987; Morrill, Mebus & Peters 1997a; Niklasson, Meadows & Peters 1984; Pepin et al. 2010). Therefore, a safe and efficacious vaccine that produces a rapid humoral response and long-term protective immunity could prevent human and animal disease and save economic resources in an outbreak situation (Morrill et al. 2013b). However, the currently used RVF vaccines have not had a significant impact on the prevention of RVF in livestock, and approved vaccines are not available for human use (Morrill & Peters 2003). Some of the promising RVF vaccine candidates being evaluated include the mutagenesis passage 12 (MP-12) vaccine and a recombinant candidate vaccine derived from MP-12, referred to as arMP-12ΔNSm21/384 (Caplen, Peters & Bishop 1985; Saluzzo & Smith 1990; Won et al. 2007). Rift Valley fever MP-12 is a live attenuated mutagenised vaccine that was developed from a virulent Egyptian RVFV strain, ZH548, by 12 serial passages in human foetal lung fibroblast (MRC-5) cells in the presence of 5-flourouracil. As a result, mutations were induced in the large, medium and small RNA segments resulting in attenuation of the virus through amino acid changes (Vialat et al. 1997). Although the MP-12 vaccine candidate was found to be safe and immunogenic in human volunteers, efforts to develop RVF MP-12 vaccine for human use were suspended because of other priorities (Ikegami & Makino 2009; Pittman et al. 2016a, 2016b). Moreover, extensive testing of the MP-12 vaccine found it to be safe and immunogenic in small laboratory animals, non-human primates, as well as in sheep and cattle (Bird et al. 2009; Morrill et al. 1987, 1991, 1997b, Morrill & Peters 2011). As a potential veterinary vaccine, MP-12 was not considered to be a promising candidate because it does not have biomarkers to distinguish naturally infected animals from vaccinated animals (DIVA). Therefore, reverse genetic technology was used to develop a recombinant vaccine (arMP-12ΔNSm21/384) that has nucleotides 21–384 deleted from the non-structural regions of the M segment to serve as a potential DIVA vaccine (Ikegami et al. 2006; Kalveram et al. 2011; Won et al. 2007).

Safety and immunogenicity studies conducted in the USA demonstrated that the arMP-12ΔNSm21/384 candidate vaccine was safe and immunogenic in sheep and calves using doses ranging from 1 × 10^3^ through 1 × 10^5^ plaque forming units (PFU) and was non-abortigenic and non-teratogenic in pregnant ewes vaccinated during the early gestation period (Morrill et al. 2013a, 2013b). Moreover, sheep vaccinated with this vaccine and then challenged with a virulent strain of RVFV were protected during experimental studies in Canada (Weingartl et al. 2014). Although MP-12 and arMP-12ΔNSm21/384 vaccine candidates have been shown to be safe and efficacious in sheep and calves in the United States (US), and the arMP-12ΔNSm21/384 vaccine in sheep in Canada, studies have not been conducted to assess the safety and immunogenicity of these vaccines in these target species or in goats in an RVFV-enzootic African country such as Tanzania. Therefore, the aim of this study was to assess safety and immunogenicity of MP-12 and arMP-12ΔNSm21/384 vaccine candidates in goats (*Capra aegagru hircus)* in Tanzania.

## Materials and methods

### Study area

Animal experiments were conducted in an insect-proof animal biosafety level 2 (ABSL-2) facility and laboratory testing of blood samples from the animals was performed in a biosafety level 2 (BSL-2) virology laboratory located at Sokoine University of Agriculture (SUA), Morogoro, Tanzania. The Morogoro district is located at latitude 6°49’S and 37°39’E with an elevation peak at 1200 m above sea level. It is bordered by seven regions: Tanga and Manyara to the north; Ruvuma, Iringa and Njombe to the south; the Coastal Region to the east; and Dodoma to the west. It has a total of eight districts, namely, Kilosa, Mvomero, Ulanga, Gairo, Kilombero, Morogoro Rural and Morogoro District.

### Experimental animals

Healthy *C. aegagrus hircus* goats 6–9 months old were used in this study. A total of 15 animals were purchased from local vendors in the Mvomero District of the Morogoro Region of Tanzania and housed in the SUA ABSL-2 facility. Prior to entering the facility, all animals were sprayed with Steladone^®^ 300 emulsifiable concentrate (EC) acaricide to remove and prevent introduction of ectoparasites. In addition, all animals were treated orally with 4 mL of 2.5% albendazole for possible parasites. The animals were individually identified using numbered ear tags and acclimatised in the facility for 2 weeks before use in the experiments. All 15 animals were housed in the same room of the facility. Throughout the experiment, all animals were given fresh grass three times a day, supplemented with maize bran, a mineral block and water ad libitum, and were observed daily for elevated body temperature as a possible indication of illness.

### Vero E6 cells and vaccine viruses

The Vero E6 cells used in this study were kindly provided by the University of Texas at El Paso (UTEP), Texas, US. Aliquots of 1.0 mL in freeze-dried form of the arMP-12ΔNSm21/384 vaccine (Lot No. 15/3/2017) were provided by the Multi-chemical Industry (MCI) Santé Animale Biopharmaceutical Company in Mohammedia, Morocco. The identity of arMP-12ΔNSm21/384 virus was confirmed at MCI using a qualitative real-time polymerase chain reaction assay (Nfon et al. 2012) targeting the L and M viral RNA segments (Morrill & Peters 2011; Njenga et al. 2015) followed by sequencing at the GENEWIZ laboratories (GENEWIZ Global Headquarters; US) using next generation sequencing technology (Illumina method: 1 × 50 bp single read HiSeq2500, High Output, per lane [V4 chemistry]). The infectivity titre of the arMP-12ΔNSm21/384 vaccine virus was 10^5.5^ tissue culture infectious dose _50__%_ (TCID_50_/mL in Vero E6 cells. The MP-12 virus was originally obtained by UTEP from the World Reference Centre for Emerging Viruses and Arboviruses, Department of Microbiology and Immunology, University of Texas Medical Branch, Galveston, Texas, USA. At UTEP, the identity of the MP-12 vaccine virus was confirmed using the plaque reduction neutralisation test (PRNT) and a RVFV MP-12-specific monoclonal antibody (Mab). The Mab neutralised the infectivity titre of the MP-12 virus from 10^6.0^ PFU/mL to 10^2.0^ PFU/mL but did not neutralise the infectivity titre of Sindbis and/or West Nile viruses. A virus stock of RVF MP-12 was prepared at UTEP with an infectivity titre of 1.4 × 10^7.0^ PFU/mL in Vero E6 cells and was stored in 0.5 mL aliquots at −80°C. Of this stock, 10 aliquots were provided to the SUA virology laboratory to prepare working virus stocks to support this study. At SUA, a working stock of the MP-12 virus was prepared in Vero E6 cells with an infectivity titre of 1 × 10^7.0^ PFU/mL.

### Experimental design and vaccination

The goats used in this study were divided into three groups: five animals for vaccination with MP-12, eight for arMP-12ΔNSm21/384 and two animals for negative controls. Each freeze-dried vial of arMP-12ΔNSm21/384 was reconstituted in 2 mL of Eagle’s minimum essential medium (EMEM) containing 4% foetal bovine serum (FBS) (Thermo Fisher Scientific, Carlsbad, CA, USA). Each reconstituted vial contained 1 × 10^5.0^ PFU/mL of the arMP-12ΔNSm21/384 virus. The MP-12 vaccine virus was diluted in EMEM to yield a concentration of 1 × 10^5.0^ PFU/mL from the initial concentration of 1.4 × 10^7.0^ PFU/mL. One millilitre of each virus was loaded into separate 5 mL syringes in a class IIA2 biosafety cabinet (NuAire, Plymouth, MN, USA) and transported in a cool box on ice to the ABSL-2 animal facility. An 18-gauge needle was attached to each of the 5 mL syringes and the animals were vaccinated intramuscularly (IM) in the neck area with 1 mL per animal. The two control animals were vaccinated likewise with 1 mL of EMEM containing 4% FBS.

### Specimen collection and preparation

Blood samples (4 mL) were collected from the jugular vein of each manually restrained goat using a 6 mL vacutainer tube. Serum (2 mL – 3 mL) was obtained from each of the animal blood samples after leaving the samples overnight at 4 °C followed by centrifugation at 1200 g for 10 minutes. Aliquots of 0.5 mL – 1.0 mL of each serum sample were transferred to sterile prelabelled vials and stored at −80 °C in an ultra-low temperature freezer until tested for RVFV and/or RVFV-neutralising antibody. Serum samples were collected 14 days before vaccination, as well as on Day 0 immediately before vaccination, and were tested for RVFV using a Vero E6 cell culture assay and for RVFV antibody using the PRNT. Samples obtained on Days 3, 4 and 5 were also tested for RVFV using the same cell culture assay; thereafter, samples obtained on Days 7, 14, 21, 28, 35, 70, 84 and 87 post-vaccination (PV) were tested to determine the neutralising antibody response using the PRNT. On Day 87 PV, all goats including the two EMEM control animals were revaccinated with 1 mL of 1 × 10^4.0^ PFU/mL of the MP-12 vaccine. All animals were observed for signs of illness and each week rectal temperatures were recorded. Blood samples were obtained on Days 7, 14 and 21 following revaccination to determine the neutralising antibody response using the PRNT, as described below.

### Rift Valley fever reverse transcription polymerase chain reaction

Prior to performing the RVF reverse transcription polymerase chain reaction (RT-PCR) assay, RNA was extracted from serum samples collected from goats on Day 14 before vaccination, on Day 0 of vaccination and on Days 3, 4, and 5 PV following the manufacturer’s instructions using the Siam^®^ Viral RNA Mini Kit (QIAGEN, Hilden, Germany). Sera samples were pooled in groups of two, and MP-12 virus positive and negative control samples were included during RNA extraction. After extraction, RNA was stored at −80 °C.

The QIAGEN One-Step RT-PCR Kit was used to test RNA samples for RVFV RNA. Primers targeting the M segment (551 bp) – RVF forward 5’TGT GAA CAA TAG GCA TTG G’3 and RVF reverse 3’GAC TAC CAG TCA GCT CAT TAC 5’ (Ibrahim et al. 1997) – were used at a concentration of 0.1 *µ*M. Mutagenesis passage 12 viral RNA was used as a positive control, and master mix (buffer) was used as a negative control in the RT-PCR assay. Thermocycler conditions were as follows: Initial cDNA synthesis at 50 °C for 30 min, PCR activation at 95 °C for 30 min, followed by 40 cycles at 95 °C for 30 seconds, 58 °C for 1 min and 72 °C for 2 min, then final extension at 72 °C for 10 min. The PCR amplicons, together with Hi-Lo™ DNA Marker (Bionexus, Inc. Oakland, CA, USA), were loaded and separated on a 1.5% agarose gel (stained with 10 *µ*l of gel red) using electrophoresis at 120 volts/20 cm for 45 min and visualised using a UV-transilluminators.

### Virus isolation

The sera samples obtained from goats on Day 14 before vaccination and on Day 0 of vaccination and samples obtained on Days 3, 4 and 5 PV were diluted 1:2 in EMEM supplemented with 4% FBS. Confluent monolayers of Vero E6 cells were propagated in 24-well plates, and each culture was inoculated in duplicate with 50 *µ*L of each serum sample. The cultures and inoculums were incubated for 1 h at 37 °C and agitated every 15 min to facilitate virus absorption. After absorption, 0.5 mL of EMEM supplemented with 4% FBS was added to each culture and incubation was continued at 37 °C with 5% CO_2_. Cultures were observed once daily for 10 days using an inverted microscope for cytopathic effect (CPE). After 10 days, all CPE-negative cultures were frozen, thawed and then passaged blindly in Vero E6 cells using the same procedure; they were again observed once daily for 10 days for CPE. Any cultures that developed CPE were harvested and stored in aliquots of 1.0 mL for further study using RT-PCR to determine if the CPE was caused by RVFV. If there was evidence of RVFV, all aliquots and any remaining cultures were destroyed by heating in an autoclave at 44.4 °C because of biosafety concern requirements that RVFV as a select agent must be kept in a BSL-3-plus laboratory.

All animals used in the vaccine trials were kept isolated and quarantined in a holding facility separate from the ABSL-2 facility, and if confirmed to be infected with RVFV they were not used any further in this study.

### Plaque reduction neutralisation test-80

Serum samples collected from the goats on Days 5, 7, 14, 21, 28, 35, 70, 84 and 87 PV and on Days 7, 14 and 21 PV following revaccination were tested for RVFV-neutralising antibody. Each serum sample was diluted 1:5 initially, followed by fourfold dilutions through 1:5120 in Hanks’ balanced salt solution supplemented with 1% HEPES (4-(2-hydroxyethyl)-1-piperazineethanesulfonic acid), penicillin and streptomycin and heat-inactivated FBS in a 96-well plate (Thermo Fisher Scientific). Each diluted test sera (75 *µ*L) was mixed with an equal volume of virus suspension containing approximately 60–80 RVFV PFUs. As a result, the final sera dilutions were 1:10, 1:40, 1:160, 1:640, 1:2560 and 1:10 240, containing virus ranging from 30 PFU to 40 PFU. The controls consisted of a mixture of an equal volume of 60–80 RVFV PFU with a 1:10 dilution of RVFV-positive antibody and a RVFV-negative antibody goat serum. The virus–serum dilution mixtures were incubated at 37 °C in the absence of CO_2_ for 1 hour. Next, Vero E6 cells were seeded in 24-well tissue culture plates and incubated for 4–5 days at 37 °C and 5% CO_2_ to provide 90% confluent monolayers. The growth media was then discarded from the Vero E6 cell monolayers and 50 *µ*L of each virus–serum dilution mixture was inoculated onto each of two wells of cell monolayers per sample. The virus positive antibody control serum mixtures were inoculated onto each of 20 culture wells and the virus-negative antibody control serum mixture was inoculated onto each of culture wells. Cultures and inocula were incubated for 1 h at 37 °C and 5% CO_2_ with agitation every 15 min. SeaKem agarose (1%) with an equal volume of 2x Eagle’s basal medium with Earle’s salt (EBME), HEPES, sodium bicarbonate, 8% FBS and 1% penicillin, streptomycin and L-glutamine (Thermo Fisher Scientific) was then prepared, and 0.5 mL was overlaid onto each cell culture. The agarose overlay was allowed to solidify and then the cultures were incubated for 2 days at 37 °C and 5% CO_2_. Each culture was then overlaid with 0.5 mL 1% agarose mixed with an equal volume of 2x EBME supplemented with 5% neutral red (Thermo Fisher Scientific) and incubated overnight at 37 °C. The plaque forming units were counted and the dilution of serum that reduced the RVF MP-12 virus dose by 80% was considered as the neutralising antibody titre.

### Clinical assessment of animals

Rectal body temperatures were recorded for each animal at the time of blood collection up to Day 35 PV. In addition, their general health status was assessed by veterinary personnel once a day and recorded. Animals that developed any sign of illness during the study were given a clinical examination by a veterinarian and samples were collected for analysis and diagnosis.

### Statistical analysis

Data analysis was performed using R statistical analysis software version 3.4.1. Analysis of the difference in antibody responses between goats vaccinated with the MP-12 or arMP-12ΔNSm21/384 vaccines during the first vaccination and after MP-12 boosting were performed using the Welch two-sample *t*-test with a significance level of *p* ≤ 0.05.

### Ethical considerations

The animal experiment was performed according to an experimental protocol reviewed and approved by the UTEP, El Paso, Texas, and the SUA IACUC (Institutional Animal Care and Use Committee) (ref # 559105-08 and SUA/CMVBS/R.1, respectively).

## Results

### Clinical assessment of the animals

**FIGURE 1:**
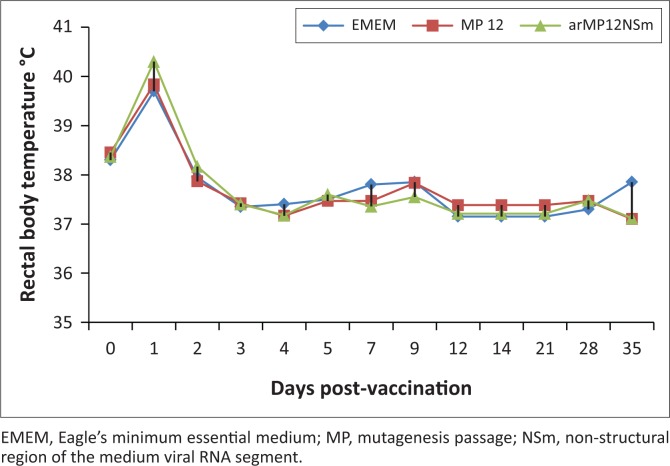
Mean rectal body temperatures of goats (*Capra aegagrus hircus*) vaccinated with Rift Valley fever MP-12 and arMP-12ΔNSm21/384 vaccines.

The rectal body temperatures of all animals before vaccination with MP-12 or arMP-12ΔNSm21/384 ranged from 38.2 °C to 38.5 °C. On Day 1 after vaccination, the temperatures of all vaccinated animals had increased to 39.0 °C, and the control animals had a temperature of 40.0 °C. On Day 2 PV and thereafter throughout the study, the temperatures of the animals ranged from 37.0 °C to 38.5 °C, including the control animals, and all animals remained healthy throughout the study (Figure 1).

### Viremia

Serum samples obtained from all goats 14 days before vaccination and on Day 0 immediately prior to vaccination with the MP-12 or arMP-12ΔNSm21/384 vaccine were negative for RVFV RNA as indicated using RT-PCR and RVFV isolation attempts in Vero E6 cells. Also, RVFV was not detected in any of the sera samples obtained on Days 0, 3, 4 and 5 PV, nor from blind passage in Vero E6 cells. Therefore, there was no detectable viremia in the goats as a result of IM vaccination with MP-12 or MP-12-NSm-del vaccines.

### Immunogenicity

All goats vaccinated with MP-12 or arMP-12ΔNSm21/384 developed neutralising antibodies; however, the two control animals inoculated with only EMEM supplemented with 4% FBS did not produce neutralising antibodies (Table 1). On Day 5 PV, all five animals vaccinated with MP-12 had neutralising antibody titres of 1:10. On Day 14 PV, three animals had neutralising titres of 1:40 and two had titres of 1:160. The antibody titres increased until Day 28 and were either sustained or decreased through to Day 87 PV, when all animals were revaccinated with 1 mL each of 1 × 10^4^ PFU/mL of the MP-12 vaccine virus. The humoral immune response in these revaccinated animals was characterised by a rapid increase in neutralising antibody titres to peak titres of 1:640 on Day 7 PV in all animals; on Day 14 titres ranged from 1:640 to 1:10 240 and on Day 21 PV from 1:2560 to 1:10 240 (Table 1).

**TABLE 1:** Rift Valley fever neutralising antibody titres in goats (*Capra aegagrus hircus*) vaccinated with 1 × 10^5.0^ plaque forming unit (PFU)/mL of Rift Valley fever MP-12 and arMP-12ΔNSm21/384 vaccine candidates and revaccinated with 1 × 10^4.0^ PFU/mL of MP-12 on Day 87 post-vaccination.

Vaccine	Animal number	-14 days?	Days post-inoculation												
			0*	5	7	14	21	28	35	70	84	87	7	14	21
EMEM	58	0	0	0	0	0	0	0	0	0	0	0	10	40	160
EMEM	112	0	0	0	0	0	0	0	0	0	0	0	40	160	160
MP-12	56	0	0	10	10	160	160	640	640	640	160	160	640	640	2560
MP-12	59	0	0	10	10	40	160	160	160	160	160	160	640	2560	2560
MP-12	60	0	0	10	40	40	160	160	640	160	160	160	640	10240	10240
MP-12	70	0	0	10	40	40	40	160	160	40	40	40	640	2560	2560
MP-12	73	0	0	10	40	160	160	640	160	160	160	160	640	2560	2560
MP-12-NSm-del	57	0	0	10	160	160	160	160	160	160	160	40	640	640	640
MP-12-NSm-del	66	0	0	0	10	10	40	40	40	40	40	40	160	160	160
MP-12-NSm-del	67	0	0	10	40	40	40	160	160	160	40	160	160	160	160
MP-12-NSm-del	68	0	0	10	40	160	160	160	640	40	40	40	640	640	2560
MP-12-NSm-del	71	0	0	0	10	40	10	40	40	160	40	40	640	640	640
MP-12-NSm-del	108	0	0	10	40	40	40	40	40	10	40	10	160	640	640
MP-12-NSm-del	110	0	0	10	40	40	40	40	160	160	160	160	640	160	160
MP-12-NSm-del	111	0	0	0	40	160	160	640	640	160	160	160	640	640	2560

Note: Data are expressed as the reciprocal of 80% plaque reduction neutralisation titre.

EMEM, Eagle’s minimum essential medium; MP, mutagenesis passage; NSm, non-structural region of the medium viral RNA segment.

† , blood samples obtained 14 days and on day 0 before vaccination

In arMP-12ΔNSm21/384 vaccinated goats, five of eight animals had neutralising antibody with titres of 1:10 on Day 5 PV, and by Day 7 PV all animals had antibody titres ranging from 1:10 to 1:160. Antibody titres remained relatively constant until Day 28, and by Day 35 a slight increase was observed in titres that were as high as 1:640 in two animals. Antibody titres then ranged from 1:40 to 1:160 until Day 87 PV. After revaccination of all animals with the MP-12 vaccine on Day 87 PV, antibody titres increased, ranging from 1:160 to 1:640 on Days 7 and 14 PV, and from 1:160 to 1:2560 on Day 21 PV. The antibody titres for the two EMEM control animals vaccinated with MP-12 and arMP-12ΔNSm21/384 were 1:10 and 1:40 on Day 7 PV, increasing to 1:160 for both animals by Day 21 PV, thus in line with the titres observed for the animals initially vaccinated with MP-12 or arMP-12ΔNSm21/384 vaccines (Table 1).

### Statistical analysis

There was no significant difference in the antibody responses between goats vaccinated with MP-12 and those vaccinated with the arMP-12ΔNSm21/384 vaccine (*p* = 0.10) during the first vaccination. However, the antibody titres for the goats that were revaccinated was significantly higher for the animals that received the MP-12 vaccine than for those that received the arMP-12ΔNSm21/384 vaccine (*p* = 0.03).

## Discussion

The results of this study indicated that the RVF MP-12 and arMP-12ΔNSm21/384 vaccine candidates elicited neutralising antibody in goats following vaccination using the IM route. Except for slightly elevated temperature of 39 °C to 40 °C on Day 1 PV, all animals maintained normal body parameters such as appetite, well-being and normal rectal temperatures ranging between 37 °C and 38 °C. The transient, slightly elevated temperatures on Day 1 PV in all animals, including the negative control animals, suggested that this observation was not related to the vaccines. The most likely reason was stress caused by manual handling of the animals during vaccination. Other virulent RVFV infection-related symptoms such as haemorrhage, diarrhoea, nasal and ocular discharge were not observed during the entire PV period. There was no evidence of virus shedding as the control animals remained negative, while being confined in the same pens with the vaccinated animals. However, further studies are needed to exclude the possibility of shedding and/or spread of the vaccine virus, including experiments designed to evaluate viral shedding in excreta, such as nasal and ocular swabs, or testing for the potential spread to highly susceptible species, such as younger or immunocompromised animals.

The RVF Smithburn and clone 13 vaccines, which are the more commonly used vaccines in Africa, especially the Smithburn vaccine, warrant concern because of a link to foetal malformations, stillbirths and abortions during the first trimester of gestation (Botros et al. 2006). Moreover, experimental studies showed that clone 13 had a potential teratogenic effect among pregnant sheep (Makoschey et al. 2016). Although this study did not assess the safety of the vaccines in pregnant goats, our preliminary results showed that both the MP-12 and arMP-12ΔNSm21/384 vaccines were safe and the antibody titres induced were considered to be high enough to protect African goats against RVFV infection. The potential protective efficacy based on antibody titres is supported by the results of a study that showed antibody titres in sheep of approximately 1:100 following vaccination with arMP-12ΔNSm21/384 vaccine were protective against challenge with a virulent strain of RVFV (Weingartl et al. 2014). Moreover, studies involving the parent MP-12 vaccine revealed that antibody titres ranging from 1:10 to 1:20 in hamsters and 1:20 in rhesus macaques afforded protection against challenge with a virulent strain of RVFV (Morrill & Peters 2011; Niklasson et al. 1984 1984).

All five goats vaccinated with MP-12 and five of eight vaccinated with arMP-12ΔNSm21/384 developed detectable neutralising antibodies by Day 5 PV, demonstrating that the vaccines elicited a rapid humoral immune response comparable to results reported for sheep inoculated with a similar dose of arMP-12ΔNSm21/384 vaccine (Morrill et al. 2013a). Moreover, the results were similar to those observed for pregnant sheep vaccinated with RVF MP-12 vaccine that developed detectable neutralising antibody from Days 5 to 7 PV (Morrill et al. 1991).

Goats vaccinated with the MP-12 vaccine developed neutralising antibodies with peak titres between 1:160 and 1:640 by Day 35 PV, which were either sustained or decreased through Day 87 PV prior to being revaccinated with the same vaccine. The rapid antibody immune response inducement and overall in increasing pattern of antibody titres suggested that the vaccine may possibly protect animals, even if administered after the onset of a RVF outbreak, as reported previously (Bird et al. 2008). In our study, a robust antibody response was observed in all goats starting from Day 7 after revaccination with the MP-12 vaccine. The antibody titres increased from 1:640 to 1:10 240 by Day 21 post-revaccination, thus suggesting that the vaccine may afford protection to animals exposed to virulent RVFV in the field.

A steady increase in neutralising antibody titres was observed in goats following vaccination with arMP-12ΔNSm21/384, with peak titres measured on Day 35 PV ranging from 1:40 to 1:640. These results demonstrated that the deletion of the non-structural region of the medium viral RNA segment (NSm) did not affect immunogenicity and that the vaccine activated B-cells and dendritic cells for initiation of antibody development. Following revaccination with the MP-12 vaccine, all goats elicited a rapid humoral immune response, and antibody titres were significantly higher than when the animals were first vaccinated, thus further demonstrating the potential of the vaccine to elicit strong immune responses in the field, if the vaccinated animals were exposed to virulent RVFV.

The antibody responses of goats following single vaccination with MP-12 or arMP-12ΔNSm21/384 did not differ significantly (*p* = 0.10), and therefore the arMP-12ΔNSm21/384, with its potential for use as a DIVA marker vaccine, could have an advantage over the MP-12 vaccine. The results were comparable to those reported for studies conducted in sheep and calves in the USA following vaccination with MP-12 and arMP-12ΔNSm21/348 (Morrill et al. 1987, 1991, 1997b, 2013a, 2013b), in which animals developed detectable neutralising antibody by Day 7 PV with a titre of 1:20. In this study, neutralising antibody were detected in most goats vaccinated with either vaccine on Day 5 PV with titres of 1:10 and in all goats on Day 7 with titres ranging from 1:10 to 1:160, slightly higher than titres reported for sheep in the USA study. The observation that sheep vaccinated with arMP-12ΔNSm21/384 developed antibody titres that were comparable to those observed for goats in this study are an indication that these animals should also be protected following challenge with virulent RVFV (Weingartl et al. 2014).

Overall, the antibody titres for goats in this study, following vaccinations with MP-12 or the arMP-12ΔNSm21/384 vaccine candidate, were slightly lower than titres observed for sheep during a study in Canada and sheep and cattle inoculated with these vaccines in the USA (Morrill et al. 1987, 1991, 1997b, 2013a, 2013b; Weingartl et al. 2014). However, the titres were comparable to those reported for goats, sheep and cattle vaccinated with RVF clone 13, despite the difference in laboratory testing procedures (Daouam et al. 2015; Dungu et al. 2010). Comparison of antibody titres among different animal species and involving different laboratories must consider possible differences in genetics, age, nutritional and health status, environment and vaccination, as well as laboratory testing procedures. Susceptibility differences may also contribute to variations among animal species in their ability to elicit immune responses to RVFV infection. For example, goats were reported to be more resistant to developing RVF disease than sheep, attributed in part to a lower and shorter viremia (Nfon et al. 2012). Therefore, the reduced amount of antigen produced in goats following vaccination, as opposed to sheep, may have resulted in a lesser amount of the vaccine virus being available to stimulate B cell secretion of antibody and may therefore have elicited a lower immune response in goats. While differences were observed in antibody titres elicited in goats vaccinated with either of the vaccines, the more critical criteria and promising feature regarding the assessment of the potential value of the MP-12 and arMP-12ΔNSm21/384 vaccines was the fact that the antibody responses were consistent with moderate and predictive protective titres. The importance of this observation is that numerous studies in the USA and Africa have demonstrated that antibodies are crucial for protection of animals against infection with RVFV (Dungu et al. 2010; Niklasson et al. 1984; Morrill & Peters 2003; Njenga et al. 2015; Pepin et al. 2010).

## Conclusion

The results of this study revealed that both the MP-12 and arMP-12ΔNSm21/384 candidate vaccines elicited the production of antibody titres to levels that could possibly afford protection to goats without inducing adverse post-vaccinal reactions. Thus, both vaccines are safe and should prove efficacious towards affording protection to this target species (goats) against virulent wild-type RVFV infection.

Other studies in progress to further evaluate the safety and immunogenicity of MP-12 and arMP-12ΔNSm21/384 in goats and sheep, as well as evaluating other routes of vaccination, such as the intradermal and intranasal routes, will provide a better understanding of the overall safety and efficacy of the candidate vaccines for use in target domestic ruminant species of RVFV in Africa.
